# A Single Resistance Exercise Session Improves Aortic Endothelial Function in Hypertensive Rats

**DOI:** 10.5935/abc.20170023

**Published:** 2017-03

**Authors:** Thaís de Oliveira Faria, Jhuli Keli Angeli, Luiz Guilherme Marchesi Mello, Gustavo Costa Pinto, Ivanita Stefanon, Dalton Valentim Vassallo, Juliana Hott de Fúcio Lizardo

**Affiliations:** Universidade Federal do Espírito Santo (UFES), Vitória, ES - Brazil

**Keywords:** Exercise, Rats, Hypertension, Nitric Oxide, Endothelium Vascular

## Abstract

**Background:**

Physical exercise is an important tool for the improvement of endothelial function.

**Objective:**

To assess the effects of acute dynamic resistance exercise on the endothelial function of spontaneously hypertensive rats (SHR).

**Methods:**

Ten minutes after exercise, the aorta was removed to evaluate the expression of endothelial nitric oxide synthase (eNOS), phosphorylated endothelial nitric oxide synthase (p-eNOS1177) and inducible nitric oxide synthase (iNOS) and to generate concentration-response curves to acetylcholine (ACh) and to phenylephrine (PHE). The PHE protocol was also performed with damaged endothelium and before and after N^G^-nitro-L-arginine methyl ester (L-NAME) and indomethacin administration. The maximal response (E_max_) and the sensitivity (EC_50_) to these drugs were evaluated.

**Results:**

ACh-induced relaxation increased in the aortic rings of exercised (Ex) rats (E_max_= -80 ± 4.6%, p < 0.05) when compared to those of controls (Ct) (E_max_ = -50 ± 6.8%). The E_max_ to PHE was decreased following exercise conditions (95 ± 7.9%, p < 0.05) when compared to control conditions (120 ± 4.2%). This response was abolished after L-NAME administration or endothelial damage. In the presence of indomethacin, the aortic rings' reactivity to PHE was decreased in both groups (EC_50_= Ex -5.9 ± 0.14 vs. Ct -6.6 ± 0.33 log µM, p < 0.05 / E_max_ = Ex 9.5 ± 2.9 vs. Ct 17 ± 6.2%, p < 0.05). Exercise did not alter the expression of eNOS and iNOS, but increased the level of p-eNOS.

**Conclusion:**

A single resistance exercise session improves endothelial function in hypertensive rats. This response seems to be mediated by increased NO production through eNOS activation.

## Introduction

The vascular endothelium has been considered to be a major target organ of arterial hypertension.^1^ Several reports have shown that endothelial dysfunction is involved in the genesis or the development of arterial hypertension and may be either the cause or the consequence of the problem.^2,3^ In the presence of arterial hypertension there is an imbalance in the production of endothelial factors, thus vasoconstrictors are produced in greater quantity than vasodilators. It explains the impaired endothelium-dependent relaxation in hypertensive animals and human subjects.^3-5^

The main cause of this endothelial dysfunction in arterial hypertension seems to be the decreased bioavailability of nitric oxide (NO).^2,3,6^ It is well known that by interacting with NO, superoxide anions (O_2_^.-^) form peroxynitrite, which decreases NO availability for smooth muscle relaxation.^7^ Endogenous inhibitors of NO synthase (NOS) are also found in the blood of hypertensive individuals, and their increased expression has been associated with greater cardiovascular risk.^3^

Physical exercise is an important tool for the improvement of endothelial function, because improves the balance between the release of vasodilators and vasoconstrictors. It has already been shown that chronic or acute exercise protocols have important effects on the release of vasoactive substances resulting in better endothelium-dependent control of vascular tone.^8-11^ Nevertheless, these data concerns endurance exercise. Thus, the effects of a single resistance exercise session on endothelial function are still poorly understood. We have previously demonstrated that a single resistance exercise session decreased the reactivity to phenylephrine (PHE) and increased the endothelium-dependent relaxation to acetylcholine (ACh) in the tail arteries of spontaneously hypertensive rats (SHR).^12^ Cheng et al.^13^ have also demonstrated similar response, however after endurance exercise.

The vascular function improvement after acute endurance exercise seems to be mediated by increased NO release.^8-11,13^ Our results suggested that acute resistance exercise also potentiates the production of that vasoactive agent, and the response was associated with the release of vasodilator prostanoids. More studies are necessary to clarify the underlying mechanisms of endothelial function after acute resistance exercise.

Thus, the present study aimed to investigate endothelial function after a single resistance exercise session in SHR.

## Methods

### Animals

The experiments were conducted using 22 male SHR that weighed 250-300 g. The rats were housed in an environment that was controlled for room temperature, humidity, light cycles (12 h light/dark). They had free access to tap water and were fed a standard rat chow ad libitum. The care and use of laboratory animals and all of the experiments were conducted in accordance with the Guide for the Care and Use of Laboratory Animals, and the protocols were approved by the Ethics Committee Escola Superior de Ciências da Santa Casa de Misericórdia de Vitória, Brazil (CEUA- EMESCAM).

### Experimental design

#### Experimental groups

The animals were submitted to surgery for direct measurement of blood pressure. All of the surgical procedures were performed using aseptic techniques. Anesthesia was induced with chloral hydrate (400 mg/kg, i.p.) and supplementary doses were administered if the rat regained a blink reflex. The left carotid artery was carefully isolated to avoid damage to any nearby nerves. A tapered polyethylene cannula (PE 50) filled with heparinized saline (100 units/ml) was inserted into the left common carotid artery for blood pressure measurement. The free end of a catheter was plugged in a stainless steel obturator and inserted subcutaneously to exit from the back of the neck. The animals were placed in separate cages and were allowed to recover for 24 hours before the initiation of the experimental procedures. The rats were monitored for any signs of infection.

Blood pressure and heart rate were continuously recorded in conscious rats before the resistance exercise session to confirm the presence of arterial hypertension. The blood pressure was determined by connecting the arterial catheter to a TSD104A pressure transducer that was coupled to a DA100C amplifier. An acquisition system (MP 100 Biopac Systems, Inc., CA, USA) was used for real-time blood pressure and heart rate recording and for subsequent analysis.

On the day of the experiment, the rats were allowed to adapt to the laboratory environment for 1 hour before their resting hemodynamic measurements were recorded. After the adaptation period, baseline blood pressure levels were measured in conscious animals for 10 minutes before exercising. Subsequently, the animals were randomly divided into two experimental groups: the exercise group (n = 11), in which rats were submitted to a single resistance exercise session; and the control group (n = 11), where the animals were submitted to a single simulation of a resistance exercise session. Ten minutes after exercise training, the animals from both groups were anesthetized with sodium thiopental (50 mg/kg, i.p.) and were euthanized by exsanguination. The thoracic aorta was carefully dissected for the analysis of vascular reactivity and protein expression.

#### Exercise protocol

Initially, all animals were adapted to the exercise apparatus for 4 - 5 days. For adaptation, the rats were placed on the exercise apparatus without weight in the rest position, and, therefore, the animals did not move, although received tail electrical stimulus. Afterwards, one repetition of the maximal test was performed. The maximum repetition (RM) was determined to be the maximum weight that was lifted by each rat using the exercise apparatus. After 2 days of rest, the animals were submitted to an exercise protocol. The rats performed the resistance exercise according to a model adapted from previous studies.^12-15^ Rats that were wearing a canvas jacket were able to regulate the twisting and flexion of their torsos and were fixed by a holder in a standing position on their hindlimbs. An electrical stimulation (20 V for 0.3-second duration and at 3-second intervals) was applied to the rat's tail through a surface electrode. As a result, the animals extended their legs repeatedly, which lifted the weight on the arm of the exercise apparatus. This apparatus was chosen because mimics traditional squat exercises that are performed by humans, and the results obtained in rat skeletal muscles are similar to those observed in humans.^15^ The rats were exercised for 20 sets with 15 repetitions per set in the exercise apparatus. The repetitions were performed at 3-second intervals with a 1-minute rest between the sets. The exercise intensity was 50% of one RM. The control group received the same stimulus for the same frequency and duration and at the same intensity and intervals as the exercise group. However, the exercise apparatus was unweighted and in the rest position, and therefore, these animals did not lift a load.

#### Vascular reactivity measurements

The thoracic aorta was carefully dissected out and cleaned of fat and connective tissue. For the reactivity experiments, the aorta was divided into 3-4 mm cylindrical segments. The functional testing of the aortic rings was performed as previously described.^16^ Briefly, 4 mm-long segments of thoracic aorta were mounted in an isolated tissue chamber containing Krebs-Henseleit solution (in mM: 118 NaCl; 4.7 KCl; 23 NaHCO_3_; 2.5 CaCl_2_; 1.2 KH_2_PO_4_; 1.2 MgSO_4_; 11 glucose and 0.01 EDTA), gassed with 95% O_2_ and 5% CO_2_, and maintained at a resting tension of 1 g at 37ºC. The isometric tension was recorded using an isometric force transducer (TSD125C, CA, USA) that was connected to an acquisition system (MP100 Biopac Systems, Inc., Santa Barbara, CA, USA).

After a 45-min equilibration period, all of the aortic rings were initially exposed twice to 75 mM KCl, the first time to check their functional integrity and the second time to assess the maximal tension that developed. Afterwards, 10 µM ACh was used to test the endothelial integrity of the segments that had been previously contracted with 1 µM PHE. A relaxation response that was equal to or greater than 90% was considered to be demonstrative of functional endothelial integrity. After a 45-min washout, concentration-response curves to PHE were determined. Single curves were generated for each segment. The role of select, local vasoactivators on the PHE-elicited contractile response was investigated. The effects of the following drugs were evaluated: (1) the nonspecific NOS inhibitor N-nitro-L-arginine methyl ester (L-NAME) (100 µM) and (2) the nonspecific cyclooxygenase (COX) inhibitor indomethacin (10 µM). These drugs were added to the bath 30 min before generating PHE concentration-response curves.

The influence of the endothelium on the response to PHE in the absence or presence of exercise was investigated after its mechanical removal was achieved by rubbing the vessel lumen with a needle. The absence of endothelium was confirmed by the inability of 10 µM ACh to induce relaxation.

In another set of experiments, after a 45-min equilibration period, the aortic rings of control and exercise rats were pre-contracted with 1 µM PHE and concentration-response curves to ACh (0.1 nM - 30 mM) were determined.

#### Western blot analyses

After performing euthanasia as previously described, the thoracic aorta was obtained. To analyze the endothelial nitric oxide synthase (eNOS) expression, phosphorylated endothelial nitric oxide synthase (p-eNOS) expression and inducible nitric oxide synthase (iNOS) expression, the arteries were rapidly frozen and kept at -80ºC. From each homogenized artery, 80 µg of protein were separated by 10% SDS-PAGE. The protein was transferred to nitrocellulose membranes that were incubated with blocking buffer, and then incubated with antibodies for eNOS, eNOS that was phosphorylated on the amino acid serine at position 1177 (p-eNOS1177) (1:250; BD Transduction Laboratories™, Lexington, UK), and iNOS (1:250; BD Transduction Laboratories™, Lexington, UK). After washing, the membranes were incubated with anti-mouse immunoglobulin antibody (1:5,000; StressGen, Victoria, Canada) that was conjugated to horseradish peroxidase. After a thorough washing, the immunocomplexes were detected using an enhanced horseradish peroxidase/luminol chemiluminescence system (ECL Plus, Amersham International, Little Chalfont, UK) and film (Hyperfilm ECL International). The signals on the immunoblot were quantified using the ImageJ computer program, and the same membrane was used to determine α-actin expression with a mouse monoclonal antibody (1:5,000; Sigma, USA).

#### Data analysis and statistics

The contractile responses were expressed as a percentage of the maximal response that was induced by 75 mM KCl. The relaxation responses to ACh were expressed as the percentage of relaxation of the maximal contractile response. For each concentration-response curve, the maximal effect (E_max_) and the concentration of agonist that produced 50% of the maximal response (-log EC_50_) were calculated using a non-linear regression analysis. Thus, the sensitivity (50% of the maximal response) of the agonists was expressed as EC_50_ (-log EC_50_) and the maximal contractile response to drug was expressed as E_max_. To compare these variables (EC_50_ and E_max_) between groups, unpaired Student's *t*-test was used.

To compare the effects of endothelial denudation or L-NAME on the contractile responses to PHE, the results were expressed as the differences in the area under the concentration-response curve (dAUC) for the control and experimental groups.

For protein expression, the data were expressed as the ratio between the signals on the immunoblot that correspond to the protein of interest and to α-actin. The differences were analyzed using unpaired Student's *t*-test. All the results were expressed as mean ± SE (standard error). *P < 0.05* was considered to be significant. For all analyses, GraphPad Prism Software (Inc., San Diego, CA, USA) was used.

## Results

### The effect of exercise on aortic reactivity

**Figure 1:**
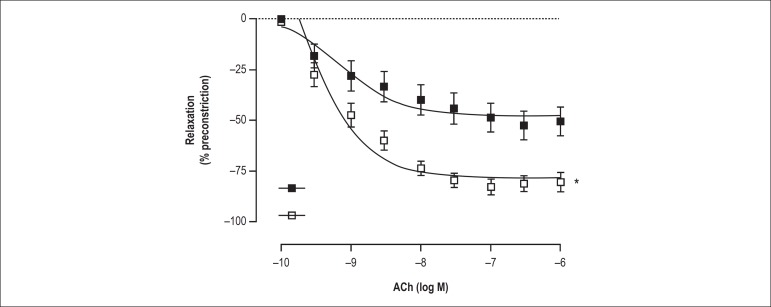
Effects of exercise training on concentration-response curve in aortic rings. Concentration-response curve to acetylcholine (ACh) obtained in aortic rings pre-contracted with phenylephrine (PHE) in control (Ct, n = 17) and exercise rats (Ex, n = 15). *p < 0.05 vs. Ct.

To investigate the attenuation of aortic reactivity after exercise, the endothelium-dependent relaxation was elicited by the addition of ACh (Figure 1). A single resistance exercise increased the endothelium-dependent relaxation as observed in the concentration-response curve to ACh. Moreover, after exercise, there was an increase in the E_max_ to ACh (p < 0.05), however, the EC_50_ was not altered (p > 0.05) (Table 1).

**Table 1:** EC_50_ and E_max_ values for each protocol

	EC_50_		E_max_	
	**Ct**	**Ex**	**Ct**	**Ex**
ACh	8.9 ± 0.2	9.0 ± 0.1	50 ± 6.8	80 ± 4.6*
E+	6.7 ± 0.09	6.7 ± 0.1	120 ± 4.2	95 ± 7.9*
E-	7.6 ± 0.1	7.7 ± 0.2	159 ± 7.2^†^	162 ± 7.1^†^
LN	7.0 ± 0.2	7.0 ± 0.2	149 ± 7.9^†^	148 ± 5.1^†^
Indo	6.6 ± 0.3	5.9 ± 0.1	17 ± 6.2^†^	9.5 ± 2.9*^†^

EC_50_: 50% of the maximum effect of the drug; E_max_: maximum effect of the drug; Ach: acetylcholine; E+: phenylephrine without damaged endothelium; E-: phenylephrine with damaged endothelium; LN: L-NAME; Indo: indomethacin on the isolated aortic rings in control (Ct, n = 11) and exercise (Ex, n = 11) conditions in spontaneously hypertensive rats;

* p < 0.05 vs. E_max_ Ct.

† p < 0.05 vs. E_max_ E+ Ct and Ex.

**Figure 2:**
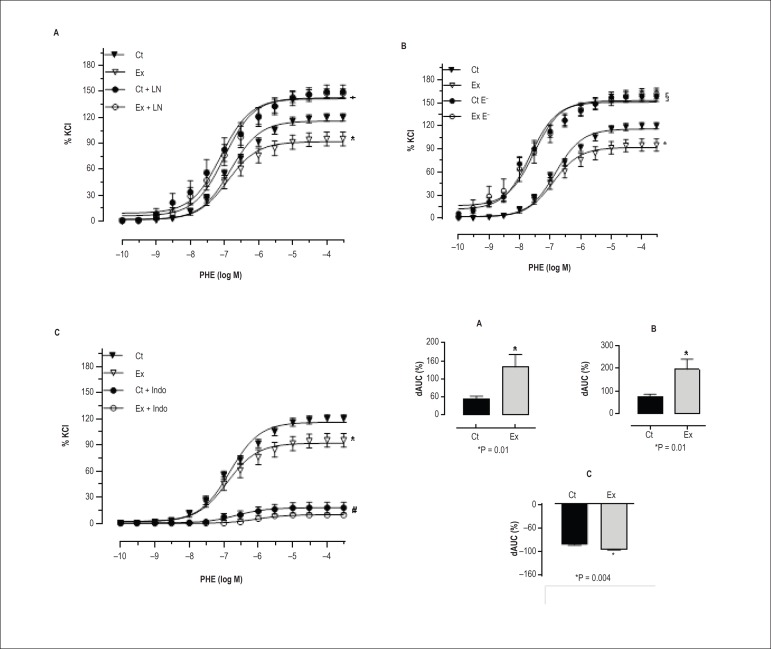
Effects of exercise training on concentration-response curve in aortic rings. Concentration-response curve to phenylephrine (PHE) obtained in aortic rings in control (Ct, n = 22) and exercise rats (Ex, n = 22) (A) before and after L-NAME administration (Ct+LN, n = 11; Ex+LN, n = 12); (B) after endothelial damage (Ct E–, n = 7; Ex E–, n = 6); and (C) after indomethacin administration (Ct+Indo, n = 7; Ex+Indo, n = 7). dAUC, difference in the area under the curve. *p < 0.05 Ex vs. other conditions. +p < 0.05 Ct+LN and Ex+LN vs. other conditions. §p < 0.05 Ct E– and Ex E– vs. other conditions. #p < 0.05 Ct+Indo and Ex+Indo vs. other conditions. The values are expressed as percentage of maximal response to KCl.

Aortic reactivity to PHE was attenuated after exercise (Table 1). In the presence of L-NAME, the decrease in vascular reactivity to PHE after exercise was abolished, and there was a significant increase in vascular response in both groups (Table 1). Figure 2 shows the concentration-response curves to PHE, as well as the percentage of the dAUC after L-NAME or indomethacin administration, and following endothelial damage. Vascular reactivity was increased significantly in both groups after endothelial damage (p < 0.05) (Figure 2; Table 1). Under this condition, the percentage change of the dAUC was also greater in the exercised rats, suggesting that there was an important endothelial modulation on the vascular reactivity to PHE (Figure 2). Aortic reactivity to PHE was significantly decreased in both groups in the presence of indomethacin, suggesting that there is an increased COX-mediated vasoconstrictor prostanoid production in hypertensive rats. This result is reinforced by the percentage change of the dAUC, which demonstrated a greater effect in the exercise group (Figure 2, Table 1).

### Expression of iNOS, eNOS and p-eNOS

**Figure 3:**
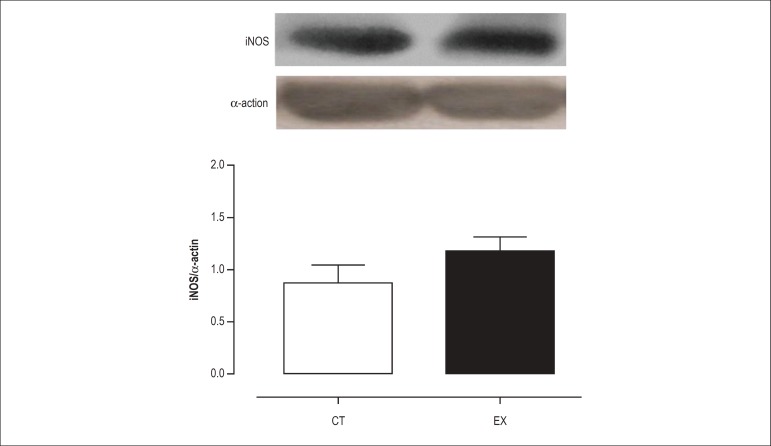
iNOS as determined by Western blot analysis in the aorta of control (Ct) and exercise training rats (Ex). Mean ± SEM (n = 7).

**Figure 4:**
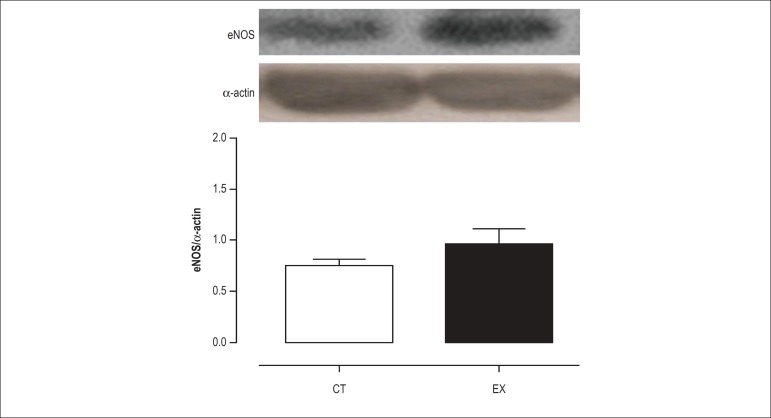
Effects of exercise on protein level. eNOS as determined by Western blot analysis in the aorta of control (Ct) and exercise training rats (Ex). Mean ± SEM (n = 7).

**Figure 5:**
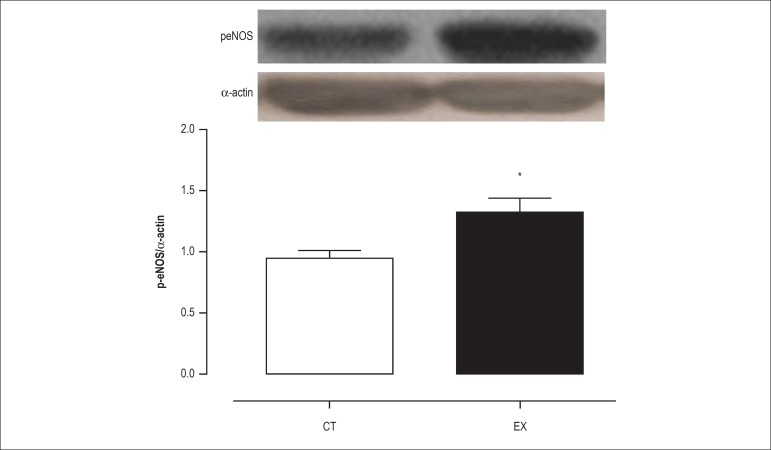
Effects of exercise on protein level. eNOS phosphorylation at Ser1177 as determined by Western blot analysis in the aorta of control (Ct) and exercise training rats (Ex). Mean ± SEM (n = 7). *p < 0.05 vs. CT.

As shown in Figures 3 and 4, the protein expression level of iNOS and eNOS was not altered after acute exercise. However, the level of p-eNOS protein was 38% higher (p < 0.05) in the exercised rats as compared to the controls (Figure 5), suggesting that there is increased NO production after a single resistance exercise session.

## Discussion

The present study demonstrated that a single resistance exercise session that was conducted at 50% of one RM increases the endothelium-mediated vasodilatation and decreases the vascular responsiveness to PHE. This response was associated with an increase in the level of p-eNOS117, indicating that NO has an important role in the improvement of endothelial function following acute exercise.

Using a similar exercise protocol, we previously demonstrated that a single resistance exercise session decreases blood pressure in conscious SHRs,^17^ reduces responsiveness to PHE and increases endothelium-dependent relaxation^12^ in the tail arteries of SHR. These responses appear to be primarily mediated by NO. It has been demonstrated that chronic exercise, as well as acute aerobic exercise, decreases α-adrenergic vascular responsiveness^18-23^ and increases endothelium-dependent relaxation in humans and in normotensive and hypertensive animals.^18,24,25^ This response is thought to be mediated by NO production and by other vasodilators, such as prostacyclin.^18,24,26^ Definitive data on the effects of acute resistance exercise on vascular function are limited. Two previous studies investigated the effects of isometric exercise using a handgrip^27,28^ and aimed to evaluate brachial artery function, primarily in patients with endothelial dysfunction. There is no other study about vascular function and acute resistance exercise in conductance vessels.

We initially evaluated the endothelium-dependent vasodilatation that was elicited by ACh on the isolated aortic rings and showed that a single resistance exercise session evoked an increase in this response (Figure 2). These results corroborate the previous findings in normotensive and hypertensive rats after acute dynamic exercise.^18-21^ Additionally, Maiorana et al.^29^ investigated the response of the brachial artery to ACh in patients with heart failure after 8 weeks of endurance and resistance training and also demonstrated a significant increase in the vasodilatation response to ACh.

We also demonstrated that acute resistance exercise decreases the vasoconstriction response to PHE mediated by increased endothelial NO production (Figure 2). Using similar methods, Howard et al.^20^ have demonstrated that a single aerobic exercise session reduced the response to PHE in normotensive rabbits. Additionally, Patil et al.^30^ have shown *in vivo* a significant attenuation of the maximal vasoconstriction response to PHE in the iliac arteries of Sprague-Dawley rats after a single running session. This response was abolished with the inhibition of NO synthesis. Similarly, in the present study, the responsiveness to PHE after exercise returned to control levels following L-NAME administration, suggesting that NO production is increased during post-exercise recovery. Rao et al.^22^ have also demonstrated a significant reduction in the responsiveness to PHE in the femoral arteries of SHR after acute exercise on a treadmill, which was abolished after the inhibition of NO synthesis with L-NAME. In humans, NO synthesis inhibition also abolishes the vasodilatation response after acute exercise.^31^ Our results are in agreement with the data obtained from chronic exercise experiments as well. Chen et al.^19^ have shown that the reduction of the vascular response to norepinephrine after treadmill training was mediated by NO. Similarly, Chen et al.^18^ has demonstrated a reduction in sensitivity to norepinephrine and PHE in the aorta of SHR and Wistar Kyoto rats after treadmill training, due to increased NO production.

Thus, it appears that both acute and chronic exercise elicit a reduction in vascular responsiveness that is primarily mediated by the increase in NO synthesis. Nevertheless, the underlying mechanisms that generate the increased NO production after chronic and acute exercise are different. It has been previously reported that exercise training increases eNOS expression.^26,32^ We investigated whether eNOS and iNOS protein expression were increased after a single resistance exercise session. As expected, the expression of these isoforms was not altered after acute exercise because it is unlikely that a single exercise session represents a sufficient stimulus to induce protein expression. Because eNOS activation is dependent on the phosphorylation pattern of well-characterized sites,^33^ we hypothesized that eNOS phosphorylation could be the mechanism involved in NO production after acute exercise; therefore, we measured p-eNOS1177 protein levels. The amino acid serine at position 1177 is the primary activating eNOS phosphorylation site, and when it is modulated by the Akt kinase (also known as kinase protein B) and eNOS, it demonstrates an increased sensitivity to baseline Ca^2+^/calmodulin concentrations.^34^ The level of p-eNOS1177 protein was significantly increased after acute resistance exercise when compared to control rats, indicating that eNOS is activated after acute resistance exercise. This finding confirms our hypothesis that the decrease of vasoconstriction and the increase of ACh-stimulated post-exercise vasodilatation were mediated by NO. Some of the factors that are involved in eNOS activation and, consequently, NO synthesis, such as shear stress, hypoxia and catecholamine release, are present during exercise and during the post-exercise recovery. Therefore, after exercise, the eNOS activity could remain increased for an extended time, resulting in a reduction in the vascular reactivity that is mediated by NO.

To investigate the possible role of vasodilator prostanoids in the reduction of vascular responsiveness after exercise, we evaluated the response to PHE in the presence of indomethacin, a COX inhibitor. In contrast to the results obtained in the tail artery in our previous study,^12^ the aortic responsiveness was significantly decreased after COX inhibition (Figure 2C) in control and exercised rats. This response may be explained by the increase of the COX-induced vasoconstrictor prostanoid production in hypertensive rats.^35^ Moreover, the increased prostanoid synthesis was shown to be due to an increase of COX-2 activity. Our data indicate that there is an important effect provoked by a single resistance exercise session. As observed in Figure 2, the percentage of the area under the curve was greater after the exercise, suggesting that acute resistance exercise decreased the vasoconstrictor prostanoid release in conductance vessels. The endothelial dysfunction present in arterial hypertension evokes an increase in vasoconstrictor prostanoid production,^35-38^ and a single resistance exercise session has an important impact on vascular function improvement, because it decreases the vasoconstrictor prostanoid release. Moreover, it is well established that NO can regulate the activity of COX enzymes^39^ and the activity of NOS is increased when the COX pathway is inhibited by indomethacin.^40^

## Conclusions

This study demonstrated that a single resistance exercise session decreased the vascular response to PHE and increased the endothelium-dependent relaxation mediated by ACh in SHR. This adaptation appears to be mediated by NO, due to the increase in the p-eNOS1117 protein levels. Moreover, the present investigation also showed that acute resistance exercise may decrease the production of vasoconstrictor prostanoids in the aortic rings of SHR. Thus, our findings suggest that the practice of resistance exercise, even in a single session, might have great clinical relevance for hypertension control.
